# Der f 31, a novel allergen from *Dermatophagoides farinae*, activates epithelial cells and enhances lung-resident group 2 innate lymphoid cells

**DOI:** 10.1038/s41598-017-04878-0

**Published:** 2017-08-17

**Authors:** Hui Wang, Jianli Lin, Lu Zeng, Chunyan Ouyang, Pixin Ran, Pingchang Yang, Zhigang Liu

**Affiliations:** 1 State Key Laboratory of Respiratory Disease for Allergy at Shenzhen University, Shenzhen Key Laboratory of Allergy & Immunology, Shenzhen University School of Medicine, Shenzhen, China; 2 Luohu District People’s Hospital, Shenzhen, China; 3 State Key Laboratory of Respiratory Disease, Guangzhou Medical College, Guangzhou, 510006 China

## Abstract

Airway epithelial cell-derived thymic stromal lymphopoietin (TSLP) and IL-33 can enhance lung-resident group 2 innate lymphoid cells (ILC2s), and they play an important role in the development of allergic diseases. This study tests the hypothesis that Der f 31 (*Dermatophagoides farinae*-31), an allergen, modulates airway epithelial cell functions and increases the frequency of lung ILC2s. Our previous research identified cofilin (Der f 31) as a novel allergen. In this study, we found that recombinant Der f 31 (r-Der f 31) upregulated the expression of co-stimulatory molecules in DCs and promoted Th2-skewed polarization. The levels of TSLP and IL-33 in epithelial cells were upregulated by r-Der f 31 via the activation of Toll-like receptor 2. Furthermore, in *in vivo* studies, r-Der f 31 induced eosinophil-like airway allergy and increased the number of lung-resident ILC2s. In summary, Der f 31 can modulate the functions of airway epithelial cells and increase levels of lung-resident ILC2s.

## Introduction

House dust mites (HDMs) are the most important source of allergens inducing airway allergies^1^. Almost 80% of asthmatic patients are sensitized to dust mites^2^. *Dermatophagoides farinae* (*D*. *farinae*) is a type of HDM that is abundant in southern China^3^. So far, 33 allergens from *D*. *farinae* have been identified. Of those identified, Der f 1 and Der f 2 are the major allergens, and nearly 87.8% patients with allergic diseases are sensitized to mite allergens^4, 5^. The treatment of allergic asthma is currently unsatisfactory. Thus, it is necessary to elucidate the mechanisms by which different allergens contribute to the pathogenesis of airway allergy.

Innate immune cells are known to play an important role in airway allergic inflammation^6^. When stimulated by allergens, lung epithelial cells can produce multiple cytokines, including thymic stromal lymphopoietin (TSLP) and IL-33^7–9^. Published data indicate that mice with high levels of TSLP are easily sensitized. Inhibiting TSLP expression in mice can attenuate the symptoms of allergic asthma^10^. Similarly, IL-33 can indirectly trigger Th2-immune responses and is important for development of allergic diseases^11, 12^. Airway hypersensitivity reactions (AHRs) and eosinophilia are reduced in IL-33-deficient mice^13^. However, factors that modulate TSLP and IL-33 expression by airway epithelial cells are not fully understood.

It is generally believed that the cytokine microenvironment plays a crucial role in Th0 cell differentiation^14^. Th2-skewed polarization is a major pathological feature of allergic diseases^15^. It is not clearly understood which cells or what cytokines initiate the skewed Th2-polarization. Recent studies indicate that group 2 innate lymphoid cells (ILC2) in the lungs can produce IL-5 and IL-13, which play an important role in Th0 differentiation^16–18^. However, the factors that activate ILC2 cells remain to be investigated.

In previous studies, *D*. *farinae* cofilin (Der f 31) was identified as a new allergen (WHO/IUIS Allergen Nomenclature Sub-committee, http://www.allergen.org/viewallergen.php?aid=816). In the present study, we sought to elucidate the role of *Der f 31* in the development of airway allergy in a mouse model, and we especially focused on the role of airway epithelial cells and lung-resident ILC2s.

## Materials and Methods

### Chemicals

PE-CD80, PE-CD83 and FITC-CD40 antibodies were purchased from Ebioscience, USA (12-0801, 12-0831 and 11-0402). Lipopolysaccharide (LPS) was purchased from Sigma, USA (L3012). Mouse GM-CSF and IL-4 were from Sino Biological, China (51048-M07H, 51084-M08B). Anti-CD3 and anti-CD28 antibodies were obtained from Ebioscience, USA (16-0031-82, 16-0281-82). Aluminum hydroxide was from Thermo Fisher, USA (77161). Peroxidase-labeled goat anti-mouse IgE, IgG1 and IgG2a Fc antibody were from Southernbiotech, USA (1110-05, 1070-05 and 1155-05). ELISA kits for IL-5 and IL-13 detection were from 4A Biotech, China (CME0003, CME0009). ELISA kits for IL-4 and IFN-γ were purchased from Ebioscience, USA (88-7044, 88-7314). Anti-mouse TLR2 antibody and Mouse IgG1, κ Isotype Ctrl were obtained from Biolegend, USA (121802, 400101). TLR4 signaling inhibitor was from Invivogen, USA (CLI-095). DNase I and collagenase D were from Sangon Biotech, China (B002004 and A004186). APC-CD45, FITC-NK-1.1, FITC-CD19 and PE-CD90 were obtained from Biolegend, USA (103111, 108705, 115505 and 205903). PerCP-IL-33R and FITC-Lineage antibodies were purchased from Ebioscience, USA (46-9333, 22-7770). PerCP-CD4, FITC-IL-4, PE-IFN-γ, PE-CD11c and PerCP-Siglec-F were purchased from Ebioscience, USA (46-0041, 11-7042, 12-7311, 12-0114 and 46-1702).

### Preparation of recombinant Der f 31 and Der f 1 (r-Der f 31 and r-Der f 1)

Synthetic *sequences of Der f 31* (GenBank accession number: KM010014) or Der f 1 (GenBank accession number: ABL84749) were ligated into a pMD19-T vector (Takara) and transformed into *E*. *coli* Top10. The target fragments were digested and ligated into PET-28a or PET-24a, then transformed into *BL21* for expression. The positive clones were induced by isopropyl-D-thiogalactopyranoside (IPTG) for 4 hours at 37 °C. The bacteria were harvested in 50 mM Tris–HCl, 100 mM NaCl, pH 7.5 and then sonicated. The target proteins were purified by affinity chromatography. The endotoxin was replaced using an ion exchange column and ToxinEraser^TM^ Endotoxin Removal Kit (L00338, Genscript, China). The concentrations of LPS tested by ToxinSensor™ Chromogenic LAL Endotoxin Assay Kit (L00350C, Genscript, China) were lower than 0.1 EU/ml.

### DC2.4 (a dendritic cell line) culture and co-stimulatory molecule detection

As we described previously^19^, DC2.4 cells (2 × 10^5^ cells/well) were seeded into 6-well dishes and maintained at 37 °C in 5% CO_2_ in Dulbecco’s Modified Eagle’s Medium (DMEM) supplemented with 10% fetal bovine serum and 10 mM HEPES(C-DMEM) overnight, then stimulated by r-Der f 31 (20 μg/ml) or LPS (1 μg/ml) for 24 hours. The cells were collected and stained with antibodies against CD80, CD40 and CD83 (Ebioscience) for 2 hours in the dark and analyzed with a flow cytometer (FACS).

### Development of airway inflammation

Four- to 7-week-old female BALB/c mice (purchased from Guangzhou Experimental Animal Center) were maintained in a pathogen-free facility, and the experimental procedures were approved by the Animal Ethic Committee at Shenzhen University. All procedures were performed according to the required guidelines. Mice were immunized subcutaneously with r-Der f 31 or r-Der f 1 (100 μg/mouse) in 0.1 ml with 2% aluminum hydroxide on days 0, 3, and 7. After one week, the mice were challenged with r-Der f 31 or r-Der f 1 (50 μg/mouse) in 50 μl PBS via nostril drop daily for one week.

The mice were sacrificed on day 22, and the middle lobes of left lung tissues were collected, fixed in 4% formalin, and embedded in paraffin wax for hematoxylin-eosin (HE) and Periodic acid–Schiff (PAS) staining. The rest of the lung tissues were cut into small pieces. The upper and inferior lobes of left lung tissues were digested with collagenase D and DNase I for 2 hours at 37 °C. The former cells were stained with CD90, Lineage, IL-33R and CD45 to detect lung-resident ILC2s by flow cytometry. The latter cells were analyzed by RT-PCR to detect the expression levels of TSLP, IL-33 and IL-13. The right lung tissues were resuspended in 500 μl PBS and processed by sonication, then the supernatants were collected to test cytokines by ELISA. In serum, the total IgE was tested by commercial ELISA kits (Ebioscience). Bronchoalveolar lavage fluid (BALF) was collected to test eosinophils by flow cytometry and optical microscopy. The levels of cytokines in BALF were measured by ELISA with commercial reagent kits (Ebioscience) according to the manufacturer’s instructions. Splenocytes were incubated in presence of r-Der f 31 or r-Der f 1 for 72 hours. The proliferation and differentiation of splenocytes were tested by FACS, and the levels of IL-4, IFN-γ and IL-10 were also detected by commercial ELISA kits (Ebioscience).

### Bone marrow-derived DCs (BMDCs)

BMDCs were generated as described previously^20^. Briefly, 4- to 7-week-old female BALB/c mice were sacrificed to obtain bone marrow cells from femurs and iliac bones, then cultured in complemented-RPMI 1640 medium with 20 ng/ml recombinant mouse GM-CSF and 10 ng/ml IL-4 for 8 days. The suspended cells were collected into 15 ml tubes by centrifuging, and washed two times by 5 ml RPMI-1640 medium to be used for further study.

### *In vitro* T-cell priming and polarization

Splenocytes were harvested from normal 4- to 7-week-old female BALB/c mice and cultured in RPMI-1640 medium with 10% FBS. Before T-cell differentiation assays, 24-wells plates were coated with anti-CD3 antibody (1 µg/ml) at 4 °C overnight and washed twice with PBS. BMDCs (2 × 10^4^) were seeded into plates and co-cultured with splenocytes (6 × 10^5^), which were stained by CSFE or not stained in the presence of r-Der f 31 (20 µg/ml) and anti-CD28 (2 µg/ml). After 3 days, the cells were collected and stained with CD4, IL-4 and IFN-γ, then assessed by FACS.

### TSLP and IL-33 in epithelial cells

A549 cells (a human epithelial cell line) were maintained at 37 °C in 5% CO_2_ in C-DMEM. A549 cells (5 × 10^5^ cells/well) were seeded into 6-well dishes and maintained at 37 °C in 5% CO_2_ overnight. In 6-well dishes, the cells were stimulated by r-Der f 31 at concentrations of 1, 5, 10 or 20 μg/ml for 4 hours, and the expression levels of IL-33 and TSLP were detected by qRT-PCR.

The right lung tissues were cut into small pieces and digested with collagenase D and DNase I for 2 hours at 37 °C. Subsequently, cells were passed through a nylon mesh sieve and then cultured in RPMI 1640 medium containing 10% FBS. The lung cells (5 × 10^6^) were seeded into 96-well plates and treated with anti-mouse TLR2 Ab or TLR4 signaling inhibitor for 120 min or 6 hours at 37 °C, respectively. Subsequently, the cells were stimulated by r-Der f 31 at concentrations of 5, 10 or 20 μg/ml. After 5 days, the supernatants were collected to test the expression levels of TSLP and IL-33.

### Quantitative real-time PCR (qRT-PCR)

An RNA extraction kit (Fastagen, China, 220010) was used to extract total RNA, and the concentration of RNA obtained was calculated by OD260 values. A total of 50 µg RNA was used for reverse transcription and RT-PCR (Transgen, China, AT341). GAPDH was used as an endogenous control, and the results were obtained from three independent replicates.

### Statistical analysis

All data are presented as the mean ± SEM and were processed by GraphPad Prism 5.0. The statistical significance between two groups was evaluated by two-tailed Student’s t-test. *P < 0.05. **P < 0.01. ***P < 0.001. ns, no significant difference.

## Results

### Der f 31 modulates the functions of DCs and induces Th2-skewed polarization

**Figure 1:**
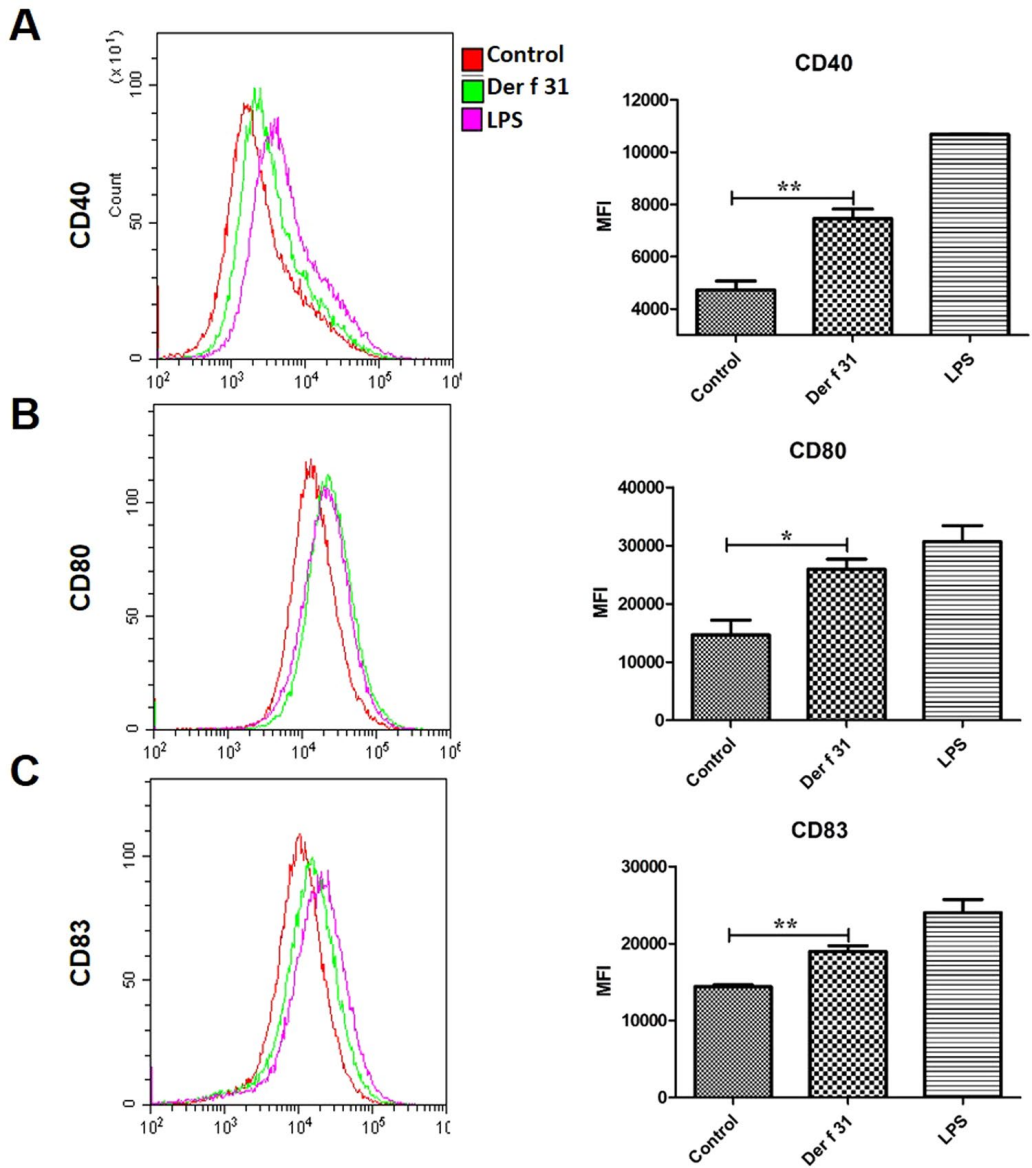
Co-stimulatory molecules are up-regulated by Der f 31. DC2.4 cells were seeded into 6-well plates for overnight culture and then stimulated with Der f 31 (20 μg/ml) or LPS (1 μg/ml) for 24 hours. The expression levels of CD40 (**A**), CD80 (**B**) and CD83 (**C**) were tested by flow cytometry. These data (means ± SEMs) were generated from three independent experiments and processed with GraphPad software. The significant differences between two groups were tested by two-tailed T-test. *p < 0.05. **p < 0.01.

**Figure 2:**
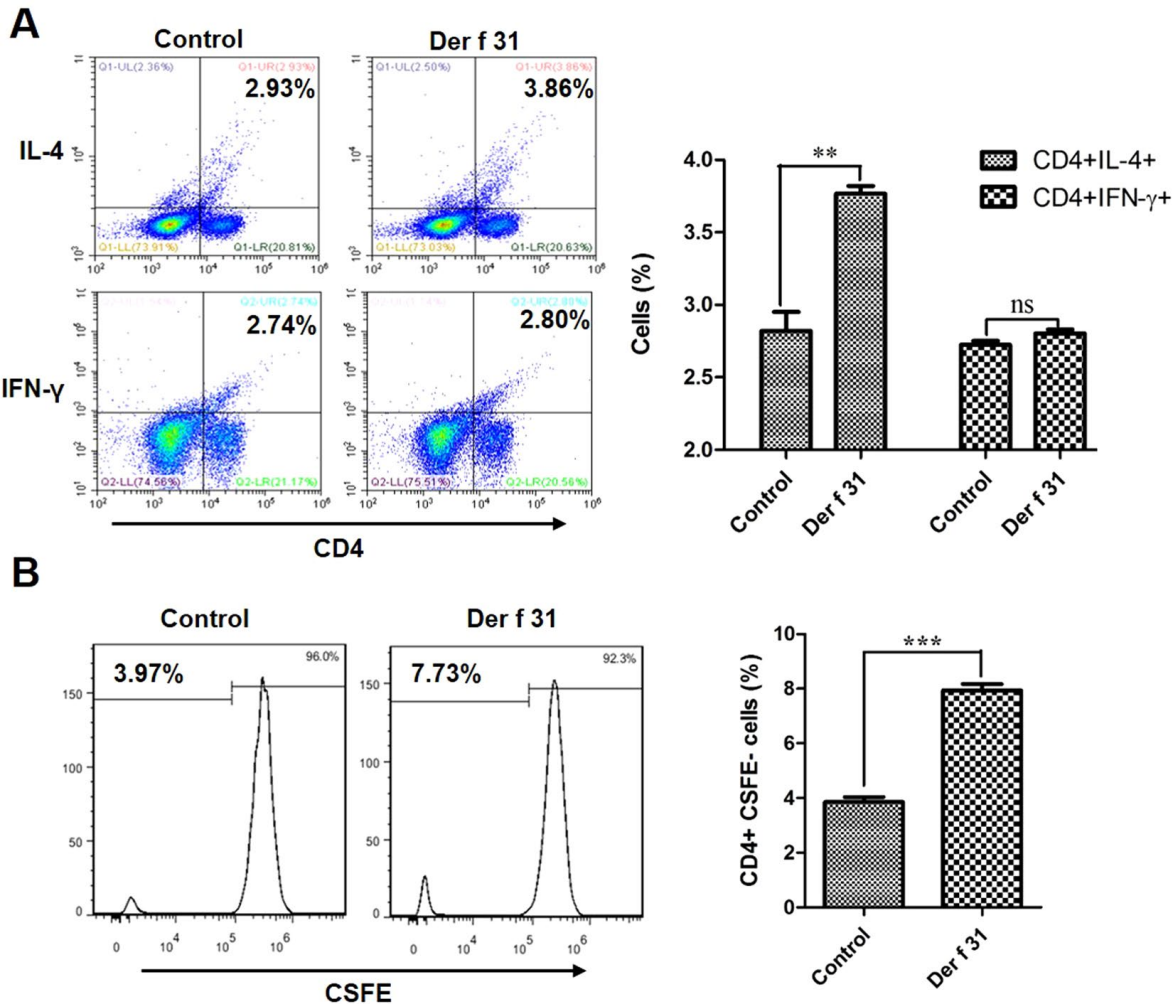
Der f 31 induces Th2-skewed polarization. BMDCs were collected from BALB/c mice, and cultured in medium with CSF2 and IL-4 for 8 days. BMDCs and spleen cells were co-cultured at 1:10 for 3 days, and proliferation and differentiation were evaluated via flow cytometry. (**A**) The differentiation of CD4+ T cells. (**B**) The proliferation of CD4+ T cells. These data (means ± SEMs) were generated from three independent experiments and processed with GraphPad software. The significant differences between two groups were tested by two-tailed T-test. **p < 0.01. ***p < 0.001. ns, no significant difference.

In our previous research, we found that Der f 31 was a new allergen of *D*. *farinae*. To further study the mechanism by which Der f 31 contributes to the initiation of airway inflammation, we observed the role of r-Der f 31 in DC phenotype and CD4+ T cell differentiation. As shown in Fig. 1A–C, r-Der f 31 increased the levels of CD40, CD80 and CD83 on DCs. BMDCs were co-cultured with spleen cells in the presence of r-Der f 31 or saline for 3 days. As shown by Fig. 2A, r-Der f 31 induced more CD4+IL4+ T cells than did the control, while the numbers of CD4+IFN-γ+ T cells did not vary. The CD4+CSFE- cells in the r-Der f 31 groups were also more abundant than in the control group, indicating that r-Der f 31 promoted proliferation of CD4+ T cells (Fig. 2B).

### TSLP and IL-33 levels from epithelial cells are regulated by Der f 31 via TLR2

**Figure 3:**
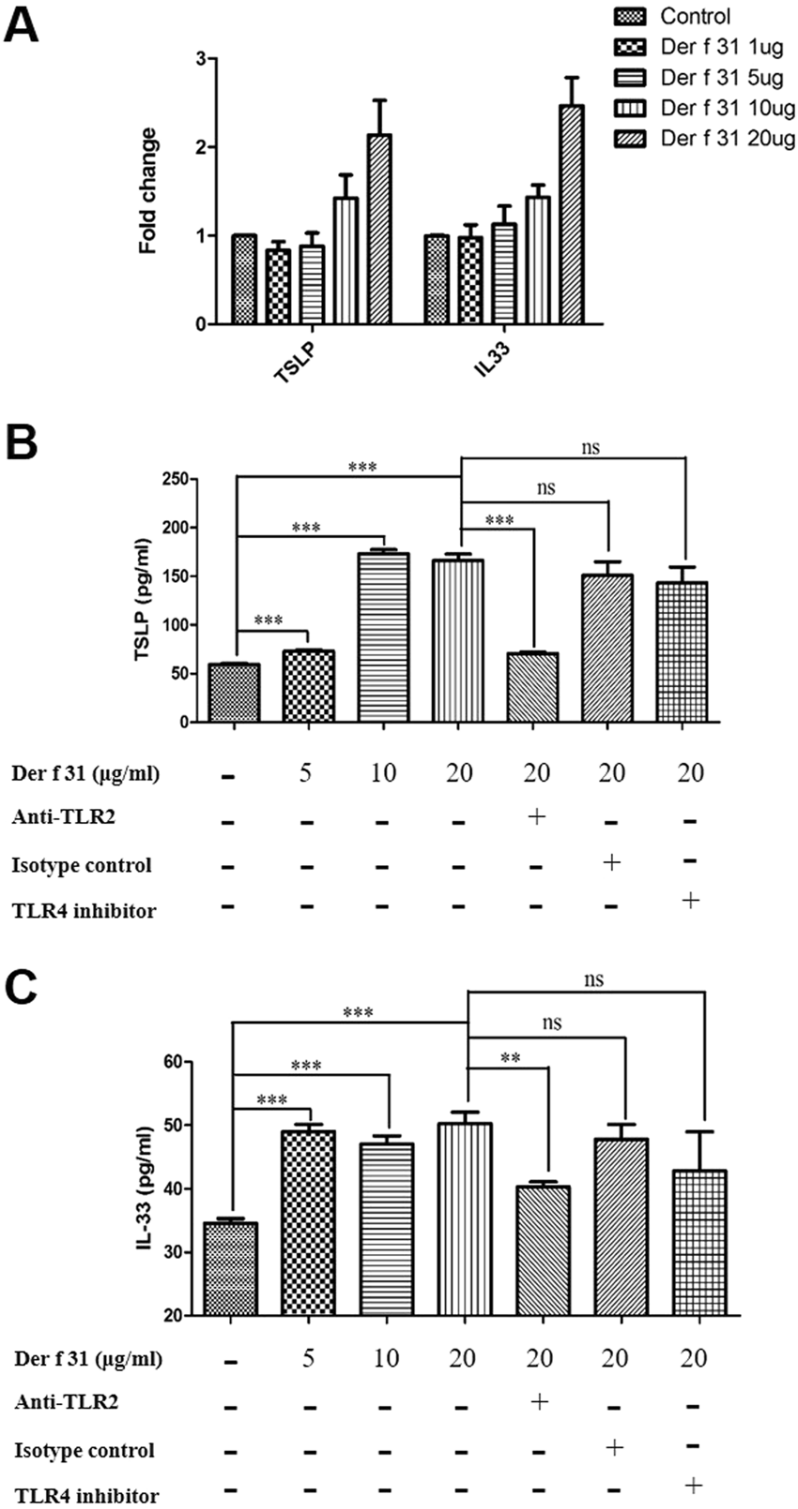
Der f 31 up-regulates the expression levels of TSLP and IL-33 in epithelial cells via TLR2. A549 cells were seeded into 6-well plates overnight and treated with different concentrations of Der f 31. At 4 hours, total RNA was extracted to analyze the expression levels of TSLP and IL-33 by RT-PCR. (**A**) The results of RT-PCR. The lung tissues from normal BALB/c mice were digested, and the cells were incubated with anti-TLR2 antibody, IgG1, κ isotype control (IC) or TLR4 inhibitor for 2 hours or 6 hours and then treated with Der f 31. At 96 hours, the supernatants were collected to perform ELISA. (**B**) The expression levels of TSLP. (**C**) The expression levels of IL-33. These data (means ± SEMs) were generated from three independent experiments and processed with GraphPad software. The significant differences between two groups were tested by two-tailed T-test. **p < 0.01. ***p < 0.001. ns, no significant difference.

It has been shown that TSLP and IL-33 are involved in the pathogenesis of asthma^21^. Therefore, we tested the levels of TSLP and IL-33 in the A549 epithelial cell line after exposure to r-Der f 31. As shown in Fig. 3A, TSLP and IL-33 from A549 cells were increased by r-Der f 31 in a concentration-dependent manner. To further study the role of Der f 31 in epithelial cells, normal lung cells were treated with r-Der f 31 for 96 hours, and the expression levels of TSLP and IL-33 were tested by ELISA. As shown by Fig. 3B,C, the levels of TSLP and IL-33 were increased by r-Der f 31 in a concentration-dependent manner, an effect that was abolished in the presence of an anti-TLR2 antibody but not a TLR4 inhibitor.

### Der f 31 induces an obvious airway allergy

**Figure 4:**
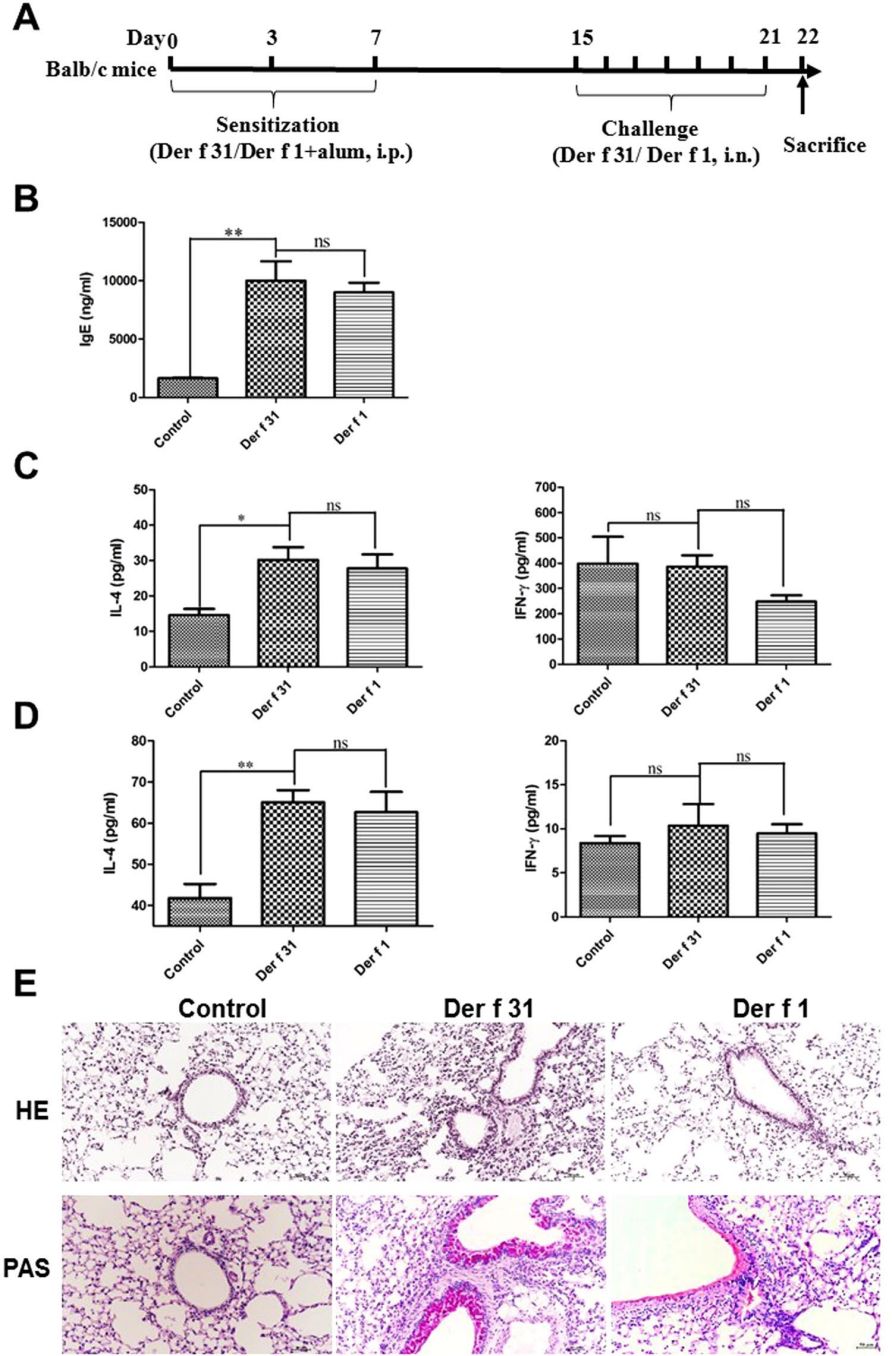
Airway allergy is induced by Der f 31. Four- to 7-week-old female BALB/c mice were immunized with PBS, Der f 31 or Der f 1 and then challenged with Der f 31 or Der f 1 for one week. (**A**) The scheme of establishment of mouse model. (**B**) Total IgE, allergen-specific IgE, IgG1 and IgG2a were detected by ELISA by optical density (OD). (**C**) The cytokines in BALF. (**D**) The cytokines in the lung homogenates. E, Histological sections of lung tissues. These data (means ± SEMs) were generated from one of three experiments (n = 6) and processed with GraphPad software. The significant differences between two groups were tested by two-tailed T-test. **p < 0.01. ***p < 0.001. ns, no significant difference.

**Figure 5:**
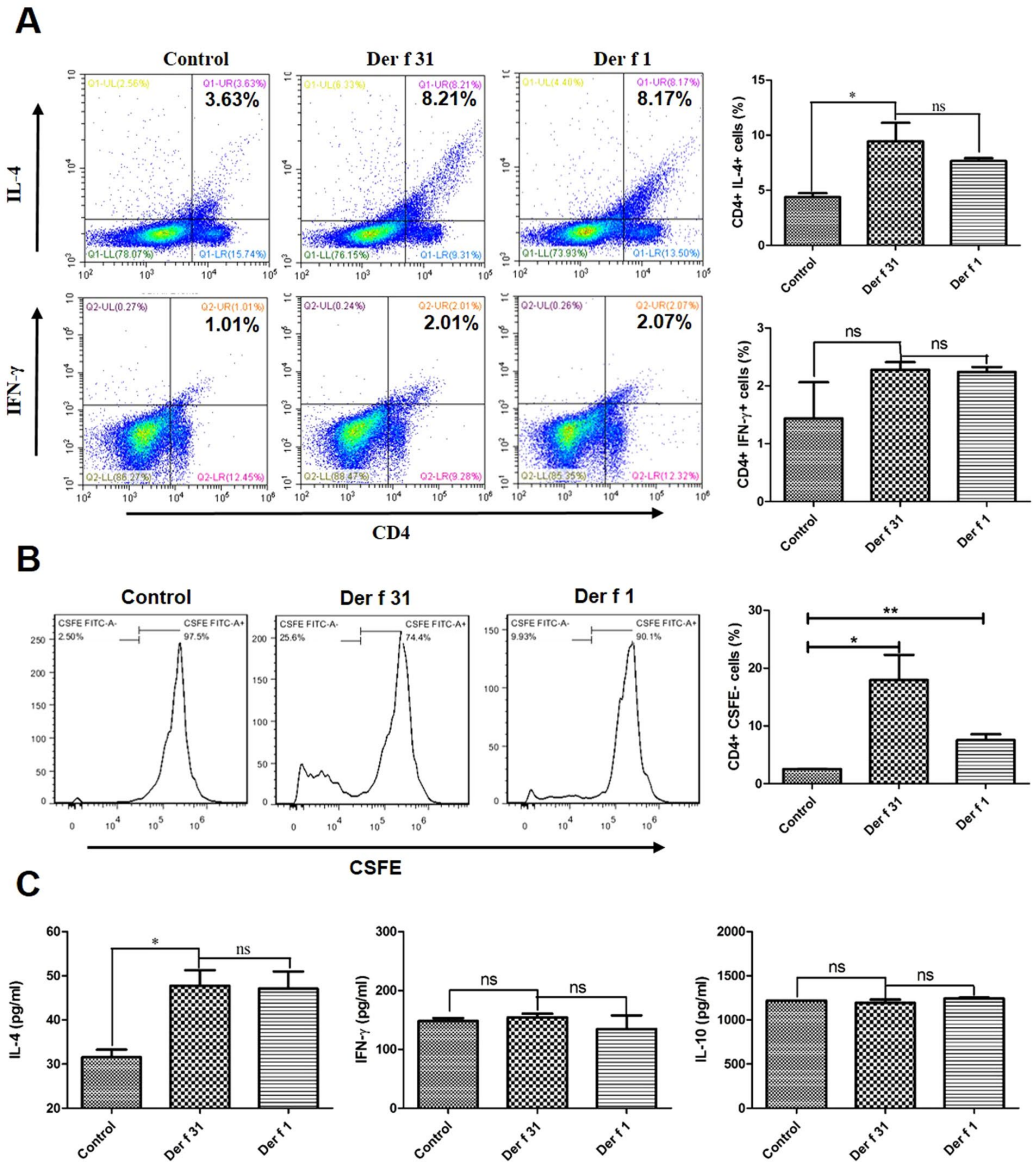
Der f 31 induces Th2-type response in spleen. The spleen cells were cultured with Der f 31 and Der f 1 for 3 days, then analyzed by FACS. (**A**) The different subtypes of T helper cells. (**B**) The proliferation of CD4+ T cells in the spleen. (**C**) The cytokines in the spleen cell supernatant. These data (means ± SEMs) were generated from one of three experiments (n = 6), processed with GraphPad software. The significant differences between two groups were tested by two-tailed T-test. **p < 0.01. ***p < 0.001. ns, no significant difference.

To elucidate the role of Der f 31 in airway inflammation, an asthmatic mouse model was developed (Fig. 4A). As shown by Fig. 4B, the serum total IgE level in the r-Der f 31 group was significantly higher than that in the control group (Fig. 4B). In the BALF, the levels of IL-4 were increased after stimulation with r-Der f 31 and r-Der f 1, but the levels of IFN-γ were not apparently changed (Fig. 4C). The levels of IL-4 and IFN-γ in lung homogenates paralleled those observed in BALF (Fig. 4D). Lung pathology and goblet cell hyperplasia were observed. As shown in Fig. 4E, r-Der f 31 and r-Der f 1-sensitized mice exhibited pathologic goblet cell hyperplasia and increased deposition of extracellular matrix in the lungs at significantly higher levels compared to the control. In the spleen, the number of CD4+IL4+ cells in the r-Der f 31 and r-Der f 1 groups was higher than that in the control group, but the number of CD4+IFN-γ+ cells was not significantly different among the groups (Fig. 5A). Furthermore, the number of CD4+CFSE- cells in the r-Der f 31 and r-Der f 1 groups was higher than that in the control group (Fig. 5B). As shown by Fig. 5C, the levels of IL-4 and IFN-γ in the supernatant of cultured spleen cells were in parallel with those in BALF, but the levels of IL-10 were not different between the groups.

### Der f 31 exposure induced airway eosinophilia as well as TSLP and IL-33 release in mice

**Figure 6:**
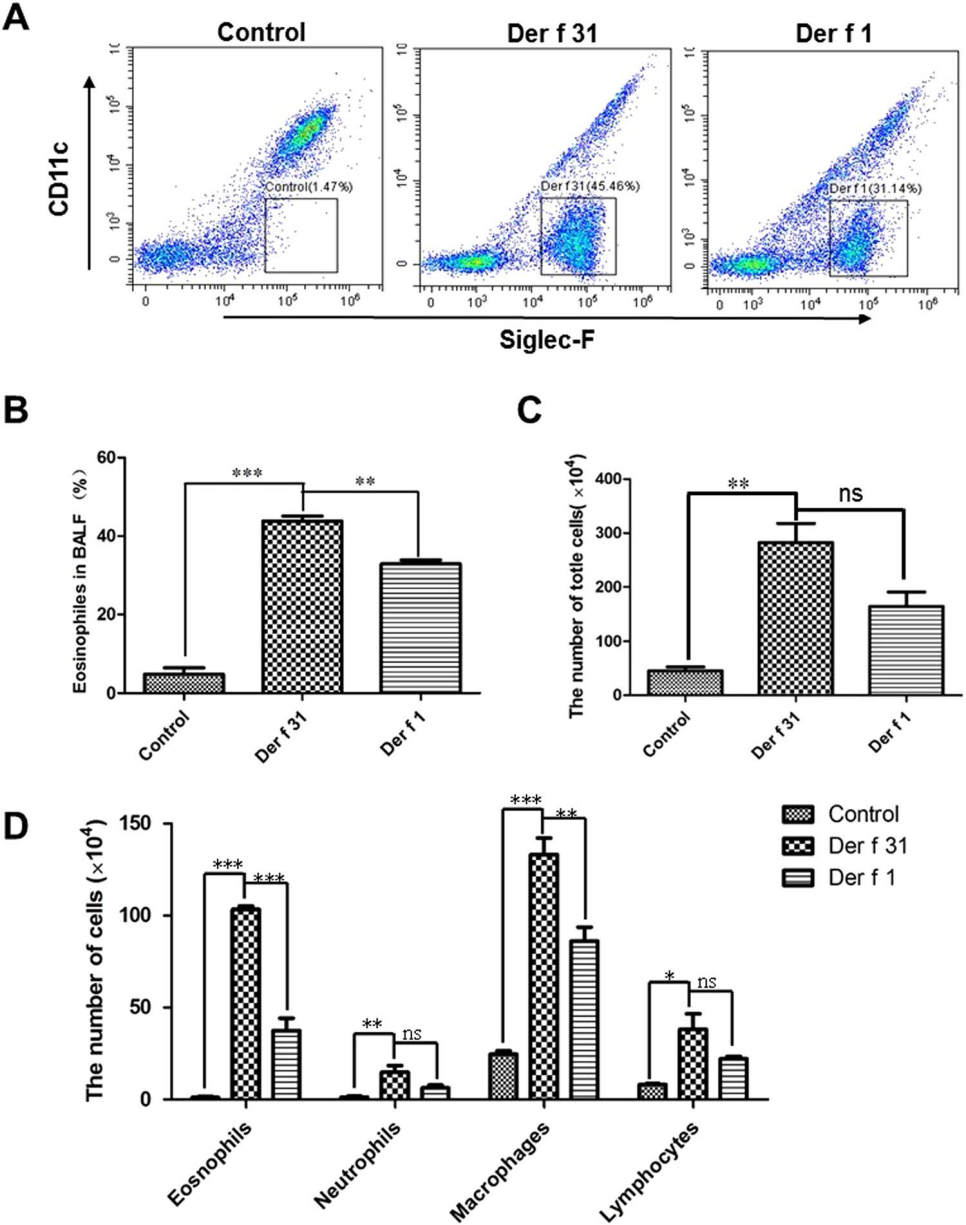
Der f 31 triggers an eosinophilia. (**A**) representative flow profile of Siglec-F+ eosinophils in BALF. (**B**) statistics chart of FACS. (**C**) the total cell counts in the BALF. (**D**) Differential cell counts in the BALF. These data (means ± SEMs) were generated from one of three experiments (n = 4–6) and processed with GraphPad software. The significant differences between two groups were tested by two-tailed T-test. **p < 0.01. ***p < 0.001. ns, no significant difference.

**Figure 7:**
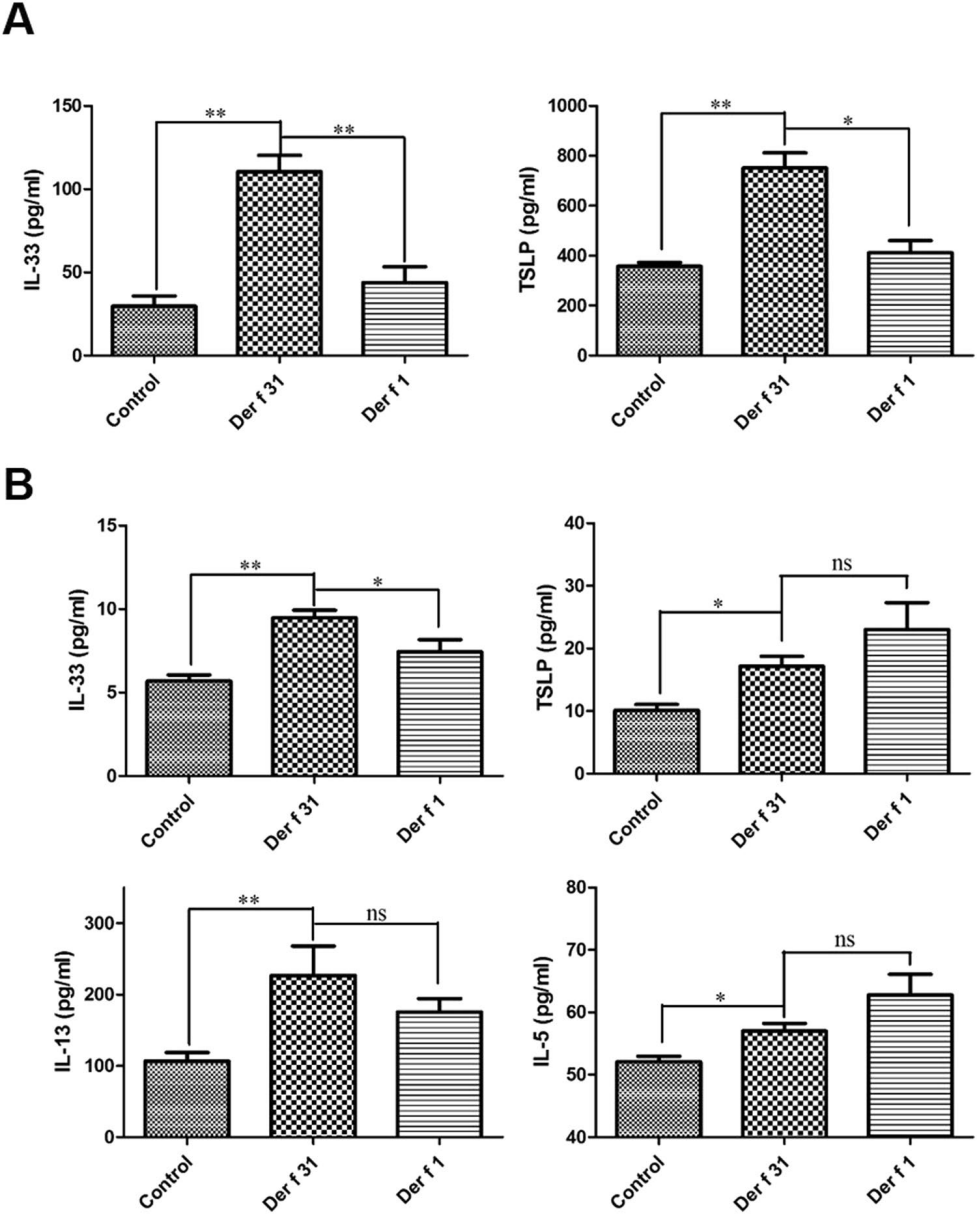
The cytokines from BALF and lung homogenates. (**A**) The cytokines in BALF. The lung tissues were homogenized by sonication on ice and then centrifuged to obtain supernatants to test the expression levels of cytokines. (**B**) The cytokines from lung homogenates. The results are means ± SEMs (n = 4–6), processed with GraphPad software. The significant differences between two groups were tested by two-tailed T-test. *p < 0.05. **p < 0.01. ***p < 0.001. ns, no significant difference.

The number and phenotypes of cells in the BALF were tested by staining and flow cytometry. As shown by Fig. 6A,B, the number of Siglec-F+CD11c- cells (eosinophils) in the r-Der f 31 and r-Der f 1 groups was higher than that in the control group, and eosinophils were more abundant in the r-Der f 31 group than in the r-Der f 1 group. As shown by Fig. 6C,D, compared to the control group, the total cells and eosinophils were markedly increased in r-Der f 31 and r-Der f 1-induced asthmatic mice, and the number of eosinophils in the r-Der f 31 group was higher than that in the r-Der f 1 group. In *in vivo* experiments, the levels of TSLP and IL-33 in the BALF and lung homogenates were higher in the r-Der f 31 group compared to the control group, and the levels in the r-Der f 31 group were higher than those in the r-Der f 1 group (Fig. 7A,B).

### Der f 31 activates lung-resident ILC2s

**Figure 8:**
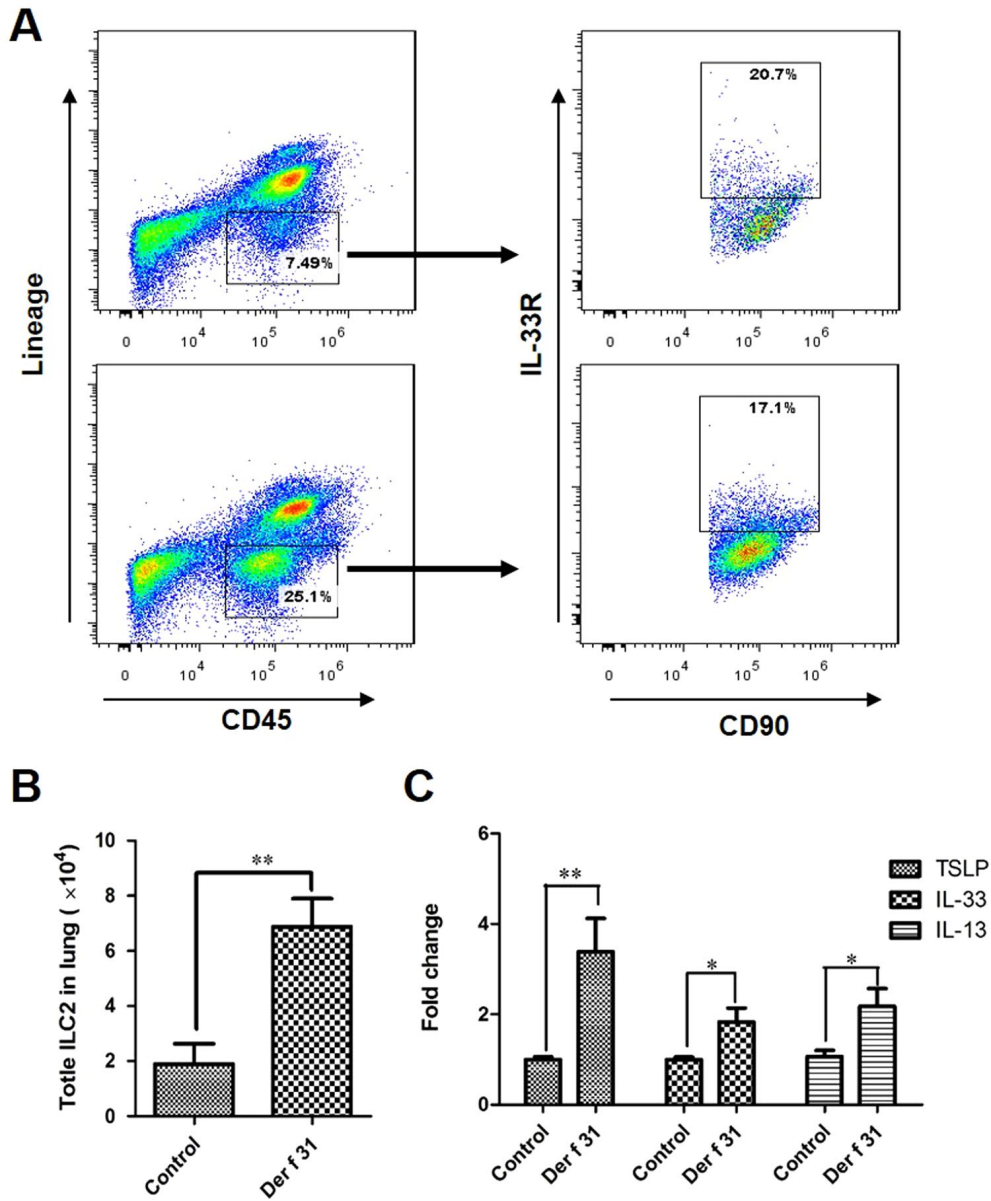
Lung-resident ILC2s are enhanced by Der f 31. (**A**) Lung ILC2s identified by FACS. (**B**) Number of lung-resident ILC2s. (**C**) The mRNA levels of TSLP, IL-33 and IL -13 in the lung. These data (Means ± SEMs) were generated from one of three experiments (n = 4–6) and processed with GraphPad software. The significant differences between two groups were tested by two-tailed T-test. *p < 0.05. **p < 0.01.

Previous reports indicated that ILC2s could be activated by epithelial cell-derived TSLP and IL-33. ILC2s mainly secrete IL-5 and IL-13, which facilitate the differentiation of CD4+ T cells^8^. We next assessed the lung ILC2s. As shown by Fig. 8A,B, the number of lung ILC2 cells was higher in the r-Der f 31 group compared to the control group. The mRNA levels of TSLP, IL-33 and IL-13 in the lung tissues were higher in the r-Der f 31 group compared to the control group (Fig. 8C). In the lung homogenates, r-Der f 31-treated mice had markedly higher IL-5 and IL-13 levels compared with those in the control group (Fig. 7B). The results suggest that Der f 31 can activate lung-resident ILC2s.

## Discussion

Previous studies have demonstrated that skewed Th2-polarization is the dominant mechanism in allergy^22, 23^. DCs are the most important antigen presenting cells (APC)^24^. Th0 cell differentiation requires not only MHC II molecules but also co-stimulatory molecules (such as CD80, CD40 and CD83)^25^. Activated Th2 cells can secrete IL-4, IL-5 and IL-13 to promote IgE production by B cells. Th2 cells activate the eosinophil and mast cells to produce cytokines and chemokines, eventually leading to the initiation of allergic asthma. Our previous research found that Der f 31 is a novel allergen in *D*. *farinae*. However, its role in allergic asthma is still unknown. In the present study, r-Der f 31 was able to modulate DCs and facilitate Th2 cell differentiation. These findings confirm that Der f 31 is a novel allergen.

Lung epithelial cells can produce a variety of cytokines, including IL-33 and TSLP^26^. These cytokines are involved in the polarization of Th2 cells and the initiation of local inflammation. At present, it is not clear which allergens modulate the expression of TSLP and IL-33 in the airway epithelial cells. In the present study, we found that r-Der f 31 up-regulated the expression of TSLP and IL-33, and this pathway was based on TLR2 but not TLR4. In *in vivo* experiments, compared to the major allergen r-Der f 1, r-Der f 31 induced higher levels of IL-33 in the BALF and lung homogenates, and higher levels of TSLP in BALF. But, TSLP in lung homogenates from r-Der f 1 and r-Der f 31 were no difference. This suggests that Der f 31 is a minor allergen that can activate the airway epithelial cells to contribute to the induction of allergic asthma.

The microenvironments of cytokines play an important role in the differentiation of Th0 cells. In addition to Th2 cells, ILC2s can also produce IL-5 and IL-13 to facilitate the development of allergic asthma^27^. In an asthmatic mouse model induced by papain, the number of lung ILC2s was significantly increased^14^. In Rag1^−/−^ mice (with ILC2 deletion), airway inflammation induced by allergens is decreased, and the phenotype is rescued by transfer of normal ILC2s^17^. Moreover, epithelium-derived cytokines containing IL-33 and TSLP can directly activate lung ILC2s^28^. Interestingly, we found that r-Der f 31 could induce TSLP and IL-33 in a concentration-dependent manner, and enhance lung ILC2s. So, we hypothesized that Der f 31 could directly modulate BMDCs to induce Th2 polarization, but the levels is lower than that *in vivo*, and the cytokines from other immune cells could enhance this process. As shown in our paper, Th2 polarization induced by r-Der f 31 is relatively low *in vitro*, but Der f 31 induced an obvious allergy *in vivo*, these evidences supported our hypothesis.

In summary, we identified epithelial cell-derived TSLP/IL-33 and lung-resident ILC2s as being responsible for airway inflammation induced by Der f 31.
